# Commentary: A lung specific escape of intravascular metastatic breast cancer cells from cytotoxic T cell killing

**DOI:** 10.3389/fimmu.2026.1793644

**Published:** 2026-02-27

**Authors:** Ling Yin

**Affiliations:** Weill Cornell Medicine, Cornell University, New York, NY, United States

**Keywords:** antigen presentation machinery (APM), interferon-gamma (IFN-γ) signaling, lung vascular niche, metastatic breast cancer, organ-specific immune evasion

Metastases are the end products of a complex, multi-step process known as the invasion-metastasis cascade, which encompasses the dissemination of cancer cells to distant organs and their subsequent adaptation to foreign tissue microenvironments (1). This lethal process is driven by the acquisition of molecular alterations within tumor cells and the co-option of stromal components, endowing metastatic cells with the traits necessary to establish macroscopic secondary tumors. A critical, yet poorly understood, facet of this adaptation is how disseminated cells evade immune destruction in specific metastatic niches. In this context, the recent study by Kizner et al. provides a compelling examination of how breast cancer cells survive within the lung vasculature by escaping cytotoxic T lymphocyte (CTL) recognition (2).

The authors employ an innovative methodological approach to address a long-standing challenge in metastasis research: visualizing rare tumor cell populations within intact organs. By utilizing three-dimensional light-sheet fluorescence microscopy (LSFM) of cleared lung lobes, they leverage a technique that functions as a non-destructive optical microtome, using a thin plane of light to achieve deep-tissue imaging with subcellular resolution while minimizing photobleaching and phototoxicity (3). This approach allows them to establish a key spatial context: OVA-expressing E0771 breast cancer cells reside predominantly inside pulmonary blood vessels. This finding directly corroborates and extends the seminal “intravascular origin of metastasis” model proposed by Al-Mehdi et al., which, through real-time epifluorescence microscopy, demonstrated that early metastatic colonies in the lung originate from tumor cells that attach to and proliferate within the vascular endothelium, rather than following a mandatory extravasation step (4). The spatial precision afforded by LSFM is thus critical for validating this model and for enabling the study’s central discovery: that these intravascular cells are not effectively engaged by circulating antigen-specific CTLs. Notably, this observation gains deeper significance in light of a contemporary study by Jakab et al., which revealed that the lung endothelium plays an active instructional role: through secretion of angiocrine Wnt factors, it directs arrested metastasizing tumor cells toward distinct fates—either intravascular proliferation or extravasation leading to latency (7). Kizner et al.’s finding that E0771 cells persist and evade immunity specifically within the vasculature aligns with the “intravascular proliferation” path defined by Jakab et al., suggesting their model may represent a subset of tumor cells pre-programmed or instructed to remain and thrive in the vascular niche.

The study’s most significant contribution lies in its mechanistic dissection of this immune escape. The authors demonstrate that E0771 cells rapidly downregulate surface expression of the specific OVA-derived peptide-MHC-I complex upon entering the lung microenvironment, while retaining MHC-I molecule expression. Transcriptomic analysis reveals this loss is driven by the coordinated downregulation of key antigen processing and presentation components, including B2M (beta-2-microglobulin) and TAP1. This mechanism resonates strongly with established findings in other cancer types. For instance, in colorectal carcinomas, total loss of MHC class I surface expression has been attributed to two major pathways: biallelic inactivation of B2M in microsatellite instability-positive (MSI+) tumors, and downregulation of antigen processing machinery (APM) components like LMP7 and TAP2 in MSI-negative tumors (5). Kizner et al. identify a similar, albeit dynamically regulated, suppression of the APM in lung-resident breast cancer cells, suggesting a convergent evasion strategy across cancers.

This mechanistic finding aligns precisely with the established role of interferon-gamma (IFN-γ) as the master transcriptional regulator of the MHC-I antigen processing machinery. As defined by Früh and Yang, IFN-γ drives the expression of proteasomal subunits (e.g., the immunoproteasome subunit PSMB8), the proteasome regulator PA28, and peptide transporters such as TAP, thereby governing the repertoire of peptides loaded onto MHC-I molecules (6). Critically, the specific downregulation of these exact IFN-γ-responsive genes—B2M, TAP1, and PSMB8—in lung-resident E0771 cells provides strong indirect evidence of impaired IFN-γ sensing or signaling within the lung vascular niche. This signaling deficit offers a compelling molecular rationale for metastatic site-specific immune evasion: the lung microenvironment may fail to provide or sustain the paracrine IFN-γ signals necessary to maintain high-level antigen presentation, rendering intravascular tumor cells transiently ‘invisible’ to circulating CTLs.

Notably, the immune evasion observed by Kizner et al. is reversible and niche-specific. E0771 cells at the primary site maintain antigen presentation and are killed by OT-I CTLs, whereas the same cells in the lungs become invisible to the same T cells. This plasticity underscores the concept that the lung vascular microenvironment actively instructs tumor cell state—a concept powerfully supported by Jakab et al., who showed that endothelial-derived signals can dictate metastatic behavior based on the tumor cell’s epigenetic predisposition (7). The findings by Kizner et al. thus add a crucial immunological dimension to this emerging paradigm of endothelial instruction. They reveal that residence within the lung vasculature, potentially guided by specific endothelial cues, not only influences proliferative versus latent fates but also confers an immune-evasive state, likely through modulation of cytokine signals like IFN-γ (6) and consequent APM dysregulation analogous to mechanisms in other cancers (5).

The translational implications are immediate and multifaceted. First, the findings explain why T cell therapies targeting primary tumor neoantigens may fail against lung metastases, as antigen presentation can be dynamically silenced in the intravascular niche. This underscores the necessity of assessing antigen presentation in metastatic sites. Second, it suggests that therapeutic strategies could aim to restore IFN-γ signaling or directly target the downregulated APM components within the metastatic niche. Third, the active role of the endothelium (4, 7) presents it as a therapeutic target; modulating endothelial-derived signals or exploiting the intravascular location of these cells (4) for drug delivery could be synergistic. Fourth, Jakab et al.’s discovery of epigenetic pre-determination (methylation status) governing responsiveness to niche signals (7) hints at the potential for patient stratification; tumors with epigenetic profiles predisposing them to intravascular proliferation and immune evasion might be identified for more aggressive or tailored combination therapies.

In summary, Kizner et al. provide a clear, mechanism-driven narrative that advances our understanding of metastatic site-specific immune evasion. By integrating advanced imaging such as LSFM (3), building upon the foundational intravascular metastasis model (4), connecting their findings to broader mechanisms of APM dysregulation (5) and its regulation by IFN-γ (6), and situating it within the contemporary framework of endothelial instruction of metastasis (7), the study offers a coherent, multi-layered, and therapeutically insightful framework. It posits that the immune evasion of lung metastases is not a passive outcome but an active adaptation, shaped by the interplay between tumor cell intrinsic pathways and instructive signals from the specialized lung vascular niche.
